# American Surgical Association

**Published:** 1888-10

**Authors:** 


### THE AMERICAN SURGICAL ASSOCIATION.

The Association was called to order by the President, Professor D. Hayes Agnew, of Philadelphia, who delivered an address on "The Relation of Social Life to Surgical Disease." He said there is no tyranny more exacting or despotic than that exercised by the conventionalities which govern our living. All stages of life from infancy to old age are under its domination. It dictates the education, the manners, the walk, the dress, the forms of speech—in fine, the whole being. Beyond all contradiction the behests of fashion are vastly more influential in governing public conduct than any arguments drawn from the teachings of structure and function. As a rule, when the conflict is between taste and reason, the victory will be on the side of taste. In nothing is this more forcibly displayed than in the apparel used to protect the body.

Let me name a few examples as illustrative of my subject. For some time the profession has been speculating on the causation of "Nasal and Post-Nasal Catarrh," with its accompanying auditory defects, the growing frequency of which cannot have escaped general observation. Doubtless no single agency will explain the presence among us of this unpleasant disease, yet there are facts connected with this affection which to me are very suggestive. I cannot recall an instance in which I have met with the disease among females belonging to the society of Friends, Dunkards, or Mennonites. If this, on more extended observation, proves to be true, may not the head-dress peculiar to these people be accepted in explanation of their exemption. The bonnet which at one time overshadowed the entire head, as all know, has been gradually shrinking in its dimensions, until it has become a mere shadow of its former self, and offers no protection whatever to the head. As a substitute, I would not insist upon the quaint headgear of the Friend, though I believe that any modification which will protect this part of the body will lessen the tendency to catarrhal inflammation of the naso-pharyngeal mucous membrane.

*Muscular Restraint.*—A legion of physical imperfections arises from muscular restraint. Among these may be mentioned weak ankles, narrow or contracted chests, round shoulders, projecting scapulæ, and lateral curvature of the spine. The foolish concession to appearance and the unwise partiality of parents for enforced systems of education, the demands of which bear no just proportion to the capacity of the infantile mind, constitute the initial or determining force of these physical imperfections. In many cases the weak ankles of children, characterized by eversion of the feet, thus allowing the superincumbent weight of the body to be transmitted to the latter inside of the proper centre of support, is largely chargeable to the miserable practice of placing on the little ones, long before they are able to walk, boots tightly laced up the limb some distance above the ankles. The confinement of the flexor and extensor muscles by this constriction prevents that free play of movement which reacts so favorably on all the elements of an articulation, and that, too, at a time when the growing forces are at full tide, so that when the time arrives for standing and walking, the muscles are unequal to the firm support of the joint. The consequence of this feebleness is soon seen in the turning outward of the feet, throwing the strain on the internal lateral ligaments, which in turn become elongated through growth, and thus the defect becomes established; but the evil does not terminate here. The calcaneo-cuboid and the astragalo-scaphoid ligaments, losing the proper support of the tendon of the posterior tibial muscle, under the abnormal tension begin to yield, and to the deformity of eversion is added that of "flat-foot." That the above is not a mere hypothetical explanation of the ankle defects I have many times verified by finding the threatening symptoms disappear after liberating the imprisoned muscles and subjecting the enfeebled parts to a judicious massage. Under no circumstances, as is too often the case, should instrumental apparatus be applied, unless in cases in which, from neglect, the deformity is thorougly established and is progressive.

Take another deformity, that of bowlegs. On the earliest signs of the unsightly curve, the limb is too often trammeled with irons, and the growth of the muscles arrested, when it is well known that if manual force be systematically applied two or three times a day the limbs will gradually assume their typical form.

Again, in further illustration of our general text, take, as an example, a child who for one long or two short sessions, for six days of the week, sits over the study desk, compelled to assume a position in which, from the inclination of the body, the shoulders fall forward, the head being supported, most probably, on the elbows and hands. In such a posture, the great serratus and pectoralis major and minor muscles are in a state of relaxation, while the erector spinæ and trapezei muscles are in a state of tension. This change in the position of the shoulders gives the scapulæ over, without antagonism or resistance, to the action of the rhomboidei and the levatores angulæ muscles, which, acting conjointly, cause that projection of the lower angle of the shoulder-blades which the older anatomists termed "scapulæ alatæ." To all this must be added the very important factor of four to six hours in the school-room, and two hours, at least, of home preparation for the following day's recitations, during which time the respiratory functions having been reduced to a minimum of activity, the muscles of the chest are comparatively passive and aëration of the blood tardy. Certainly no combination of conditions could be better devised for forming contracted chests and round shoulders. It is not long before the watchful eye of the mother detects the change in the figure of her child. She will probably discover this and take alarm, even when the pale face, the languid air, and the capricious appetite of the child cause no anxiety; and then comes the second act in the drama of physical deterioration, namely, a resort to shoulder braces and stays, in order to accomplish that which the muscles should be taught to do without restraint or incumbrance.

*Lateral Curvature.*—While it is true that lateral curvature of the spine depends upon causes both central and peripheral, yet in no small number the deformity is clearly attributable to influences of a social nature. The young column, by reason of the non-union of the epiphysis and diaphyses, and the supple character of its ligaments, is extremely flexible. Whatever, therefore, destroys the muscular equipoise, however inconsiderable the force, if persistently repeated, changes the centre of gravity and develops primary and compensating curves. For six months in the year, any fine morning, groups of young children may be seen plodding along our streets with a miniature library of books suspended from one shoulder. To the already preponderating scale of the balance, add the additional factor, a probably badly arranged light, compelling these little *savants* to assume a lateral inclination of the body in order to obtain the necessary illumination of the subjects of the study, and you have all of the conditions necessary for perpetuating the lateral deformity.

*Bodily Constriction.*—In the further discussion of my subject, I may next notice the evils of visceral displacement and pressure consequent on abdominal constriction.

The evil effects of this constriction on the viscera of the abdomen and pelvis is most strikingly witnessed in the embarrassed portal circulation, in the different uterine displacements, elongation of ligaments, displaced ovaries, tubal inflammations, hæmorrhoids, hernia and other morbid conditions which either prevent or disqualify the woman for the exercise of those functions of maternity, and which, in addition, through reflex influences, entail a host of functional disorders, reaching into every avenue of the body and invading both the mental and moral constitution of the victim. So prolific have these infirmities become that a new department of surgery has been organized for their special management. To what, if not to social causes, can these morbid changes of structure in the pelvic organs, especially of the uterus and its appendages, be attributed? Why should laceration of the cervix uteri be so common an accident? Labor is a natural process, and ought not, under ordinary circumstances, to be attended by lesion of uterine tissue. I can conceive of no agency more likely to induce that muscular degeneration which predisposes to this accident than the modes and methods of modern living, especially among the inhabitants of great cities. In the expression "modern living," much is embraced. It includes culinary pharmacy, overfeeding and drinking, insufficient or injudicious exercise, improperly heated apartments, and a disproportion between the hours of exercise and rest.

Contrast, if you will, the muscles of the hardy, country housewife, who, bearing the cares and responsibilities of a dependent family, bustles about the live-long day in-doors and out-of-doors, eats with a relish her plain and simple fare, repairs at reasonable hours to bed, and sleeps the sleep of the beloved, undisturbed by dyspeptic nightmares, and rising with the golden dawn, resumes the round of domestic toil with a clear head and supple limbs; I say, contrast this type of a class with that of another, the woman born to luxury and ease, whose capricious and exacting taste taxes the art of the professional caterer, who drags out the morning hours toying with some crazy piece of embroidery or trashy novel, lunches at one, rides out in the afternoon for an airing of two or three hours, returns to a dinner of five or six courses at seven, completes the evening at the opera, the theatre, or the assembly, and coming home after midnight, crawls into bed weary and exhausted in body and mind, only to rise, with the best hours of the morning gone, for another day of aimless routine life. Can it be doubted that in the first case, with a digestion unimpaired, with the products of textural change consumed by functional activity and eliminated through the proper emunctories, the woman should possess a vital resistance and a tone of tissue altogether superior to that of the other, whose habits of living must necessarily favor their faulty metamorphosis?

To these same agencies must be attributed that brood of nervous and hysterical evils, for the relief of which the gynæcologist too often, I fear, invades the domain of womanhood, around which her whole sexual nature revolves, and which, save only in the direst extremity, should be sacred against all operative intrusion.

*Late Marriages* constitute another social evil, the penal inflictions of which involve both sexes alike. Pride and luxury determine long engagements or deferred proposals. Marriage, it is believed, necessarily involves an establishment, a display, a retinue of servitors. The good old notion of two souls being united in wedlock for the purpose of being mutual helpmates and patiently, together, working up from modest beginnings to affluence, seems to be entirely at variance with the modern idea of this relation. In the meantime the young man is betrayed into unlawful sources of gratification, alike destructive to moral and physical purity, the pollution of which incontinence is often subsequently communicated to and perpetuated in wife and offspring. I would not dare to say how many cases of this nature had been intrusted to my professional confidence, though I doubt not my experience does not differ from that of many of my professional brethren whom I address. It is under such circumstances that many of those infective inflammations of the Fallopian tubes, as salpingitis and pyosalpinx, arise, which entail the most serious deterioration of health.

*The Foot and the Shoe.*—It may be thought by some persons that the subject of the foot and the shoe is not of sufficient dignity to appear in a public address. The Romans and the Greeks thought differently. The literature of both people is full of references to the shoe worn by both sexes. So important, indeed, are the feet to the well-being of the body that whatever impairs their usefulness, either for support or locomotion, becomes a positive calamity. Nothing can be more unlike the human foot than the modern shoe. Let anyone leave the impress of his or her foot in the wet sand of the seashore, and then place alongside of the imprint a fashionable shoe; that the two were ever intended for each other would scarcely strike a child of the forests. The North American Indian entertains juster notions about clothing this portion of his body than does the civilized denizen of New York or Philadelphia.

*Games and Amusements* which in themselves are proper and praiseworthy, too often become developed into a craze, working both moral and physical mischief. Professor Leuf, himself a professional in the national game of base-ball, has described the "pitcher's arm," a condition of over-taxed function, and one in which all the anatomical elements of the upper arm are involved. There is also the tennis arm, and the swollen supersensitive prostate of the bicyclist, both due to the abuse of popular amusements.

*Defects of Refraction*, or visual defects, constitute another class of affections fairly attributable, in many instances, to social influences. The number of children which may be seen in our streets any day wearing glasses has become a matter of common observation. It is far from being probable that the most exquisite piece of mechanism, the human eye, came from the Divine Artificer imperfect. Because eyes are young, it does not follow that they are thereby better fitted to sustain prolonged use. Just the reverse is true, and it is high time that parents and educators begin to recognize the fact. The power of the eyes for continued use, like that of other organs of the body, is one of gradation. It moves in the general procession and strengthens with the advance in life, until development has attained its zenith. Not only so, but the eye being a part of the body it must suffer or rejoice through the operation of general causes. A bone may have its normal curves changed, a tendon may slip from its appointed groove, or a blood-vessel be destroyed, and yet very little disability be realized, but the eye is made up of such extremely delicate structures, and acts according to fixed physical laws, that not the slightest alteration of a curve, or the mobility or density of its media, can occur without great vitiation of function. To exact, therefore, long hours of study from children of a tender age, involves a degree of functional strain altogether disproportionate to the structural resources of the organ, and, by disturbing the orderly processes of nutrition, gives rise to hypermetropia, asthenopia, astigmatism, and its companion, headache. That the picture is not too highly colored, or the sensation over-strained, we have only to contrast the children born and reared in those portions of the country not too much dominated by the methods of modern civilization, and who rarely demand a resort to artificial aids to provide for abnormalities of vision. The only remedy for the evil, where infantile scholarship is insisted upon, is the Kindergarten, or object system, the most natural and effective plan of impressing the young mind.

*Renal Disease.*—Is there any reasonable explanation drawn from sources of a social nature for the great frequency of these renal disorders, which come more particularly under the care of the surgeon, as crystalline deposits and calculi? For maintaining the general health at the highest physiological standard, a proper quality of food and the proper disposal of tissue waste are essential conditions. Along with wealth and luxury come the abuses of the table. Americans are fast becoming a nation of dyspeptics. Our country is so rich in the products of every zone, that nowhere else in the world can you find such a variety of foods, animal and vegetable. These foods, manipulated in a thousand ways by the subtle art of the professional cook, almost necessarily betray one into excess, and also create the desire for wine and other alcoholic beverages to aid the stomach in disposing of its plethoric supply.

In great cities, which furnish relatively the largest number of cases of renal disease, affecting pre-eminently the mercantile and sedentary classes, we find just the conditions favorable to their development. The competitions of trade keep the merchant always at white heat. Time is golden, and the street-car and other means of conveyance annihilate distance, and the ride is substituted for the needful walk. A hasty lunch at the most convenient restaurant satisfies the inner man until the business of the day is closed, when, weary and worn, he is driven to his home to partake of a course dinner, the balance of the evening to be spent on the lounge with the evening paper, or the latest periodical. To the literary man the fascinations of the study and the library charm him away, with their siren voices, from the fields and the highways, until bodily exercise grows distasteful and repugnant. In the meantime there has been no provision made for the waste or tissue metamorphoses of the body through that great agency, exercise. These accumulate in the blood, the internal eliminating organs, of which the kidneys are chief, are overtaxed, and then follow the evils of malassimilation and of excretion, in the form of urates and oxalates, often resulting in the formation of calculi.

In conclusion, may we ever hope for a time when the race will realize that these bodies which we wear, which God has so highly honored by his own incarnation, are sacred temples to be kept in harmony with recognized physical laws, and not to be made instruments of mere animal gratification.

Professor JOHN ASHHURST, of Philadelphia, read a paper entitled "A Contribution to the Study of Excisions of the larger Joints."

It was based upon the records of 120 cases occurring in his own practice, in which excision of the larger joints had been required, embracing ten of shoulder-joint; nineteen of elbow-joint; forty of hip-joint; fifty-one of knee-joint, and six of ankle-joint excisions.

The large majority of the excisions had been performed without any of the so-called "antiseptic precautions," and the wounds had been dressed with simple oiled lint, or with lint saturated with dilute alcohol. For more than a year past, however, he had employed the antiseptic method in almost all his large operations, using also antiseptic dressings in their after-treatment, and with benefit; though as regards the ultimate welfare of the patients he has not noticed any gain. His best series of consecutive successes had been obtained under old methods, and he had not obtained any diminution of mortality by the adoption of the new. At the same time he had seen no ill results which could be attributed to the use of antiseptic measures; their use shortens the period of convalescence, and they have the merit, on account of the infrequent change of dressings needed, that they greatly lessen the surgeon's labor.

Professor F. S. DENNIS, of New York, called attention to cases of excision of knee-joint for disease beginning in abscess in the condyle. In these cases the abscess cavity breaks down after recovery, leading to the production of deformity. In these cases he takes away all of the abscess-cavity, saws away a corresponding piece from the tibia, and brings the oblique surfaces together.

He has been in the habit of employing antiseptic dressings, removing the drainage-tube on the third day, allowing the first dressing to remain five or six weeks.

In excision for injury, only enough bone to allow of free drainage should be removed.

Sir WILLIAM MACCORMAC, of London, England, had heard with some surprise that the tendency seemed to be to postpone excision of hip-joint until all other measures had failed. This is not the practice in regard to any other joint. In England the disposition is to perform the operation at an earlier period. Another point that he had not heard mentioned in the paper was in reference to the performance of operation in cases of old dislocation of the joint. He had performed this operation with success in old hip-joint dislocation, and reported the case of a sailor coming under observation three years after the occurrence of dislocation of hip-joint which had not been reduced. After the operation he could use the limb perfectly. He had been much interested in the recommendation of Dr. Ashhurst that a long incision be made to reach the subcrural bursa, a suggestion which he had not heard mentioned by any other surgeon.

Dr. E. M. MOORE, of Rochester, had been somewhat surprised to hear a certain amount of indifference expressed toward the use of antiseptic surgery. He had found in his practice the greatest improvement follow the use of antiseptic dressing in these cases of excision. He cited several cases showing the result obtained.

Professor JOHN E. OWENS, of Chicago, agreed to the value of the wire cuirass, and referred to a modification of this apparatus, consisting in the substitution of a frame of gas-pipe conforming to the outlines of the body. On this the body is supported by means of flannel stretched between the two sides of the frames. Extension may be applied, if desired, by the use of adhesive plaster, counter-extension being provided for by elevating the foot of the frame. After keeping patients in bed for thirty days, he tries to get them into the open air. He had found great advantage in keeping up a certain amount of extension after the patient was allowed to get up. By removing pain this enables the patient to move the joint more freely, and thus tends to far greater mobility of the part. He thought that there was no comparison between the antiseptic methods and those formerly employed.

Dr. F. LANGE, of New York, referred to a class of cases in which the disease of the hip began in the tissue outside of the joint, the articulation becoming involved at a later stage of the affection. In these cases he recommended early operation with the hope that in this way necessity for opening the joint would be avoided.

Professor NICHOLAS SENN, of Milwaukee, read a paper on "The Relation of Microörganism to Injuries and Surgical Diseases."

At the present time no argument is required to show that many special conditions are due to the presence of bacteria. In regard to the so-called hereditary transmission of disease, the author held that the specific microbes of the specific diseases are transmitted directly from parent to child. In evidence of this he referred to cases of so-called hereditary osteomyelitis in newly-born infants. In other cases, while this same origin may be inferred, we have as yet no direct evidence that such is the case. In regard to the question whether or not pathogenic micro-organisms exist in the healthy body, while the results of some observers point in this direction, the results of others are opposed to the existence of pathogenic organisms in the healthy body. The conclusion was that under certain circumstances pathogenic organisms might be present. There is proof of this in cases in which, after accidental injury, there is localization of these pathogenic organisms. Acute suppurative infectious osteomyelitis, following slight injury or exposure, was cited as an illustration of this fact. This localization is favored by certain anatomical conditions. The antagonism among micro-organisms was being considered when the time of the author expired.

Professor SENN, in concluding the discussion, said that the diseases enumerated in his paper included only those in which the specific cause had been isolated, cultivated outside of the human body, and in which the injection of this culture produces identical lesions. When these three things are done we have furnished positive proof that the disease is due to specific germs. Another class of diseases had been alluded to in the paper in which there was reason to believe from analogy that the affection was due to specific germs, although the three conditions above referred to had not as yet been fulfilled. So far no one had been able to show that the supposed bacillus of syphilis was the specific bacillus. That it is a specific disease can not be doubted; that it is due to a microbe can not be doubted, but to establish this positively, experimenters must do what Koch did before he announced the specific origin of tuberculosis.

He was firmly convinced from his observations that tumors in the true sense of the word were not due to microbes. He had made tumor implantations for many years in animals, and in justifiable cases in man, both close to the original seat of disease and at remote points, without obtaining the least evidence of the *microbic origin* of disease.

Professor W. W. Keen, of Philadelphia, then read a paper entitled "Three Cases of Brain Surgery."

The first case was one of removal of a large tumor from the brain; the second, trephining for old depressed fracture followed by epilepsy, with removal of underlying brain substance; the third was the removal of the cerebral motor centre for the left wrist and hand, for epilepsy.

Professor N. Senn, of Milwaukee, read a paper on the "Relation of Micro-Organisms to Injuries and Surgical Diseases." The conclusion was that, under certain circumstances, pathogenic organisms might be present. Acute suppurative infectious osteo-myelitis, following slight injury or exposure, was cited as an illustration of this fact.

Professor Roswell Park, of Buffalo, had examined pus from fifty-two sources, and presented a table showing the number of cases in which pyogenic bacteria were found.

### Second Day.

Dr. Hunter McGuire, of Richmond, Virginia, read a paper on "The Formation of an Artificial Uretha for Prostatic Obstruction."

It has been my lot to meet with a number of cases of hypertrophy of the prostate gland, which produce more or less obstruction to the passage of the urine. These are conveniently divided into three classes.

1. Cases where the obstruction was due to temporary congestion of an already enlarged gland, which yielded to the ordinary treatment and did not return.

2. A class of cases when the obstruction to the passage of urine was permanent but not great. Attention to the general health, the occasional introduction of the catheter and washing out the bladder were all that the cases required. These cases are, however, never free from danger from exposure, etc, and gradual enlargement may go on and bring about the condition met with in the third class.

3. In these cases the obstruction is great and fixed; micturition is frequent and difficult, perhaps impossible without the aid of the catheter. The introduction of the instrument grows more and more difficult. Offensive residual urine is always present, and the general health suffers greatly. Cystitis, localized or general, is a painful and pronounced symptom. Violent tenesmus of the bladder, provoked by the obstruction, injures the vesical ends of the ureters, possibly a reflux of stale urine is driven into these canals, and ureteritis follows, then pyelitis and pyelonephrosis, from which the patient dies.

The paper was devoted to a consideration of surgical interference in this third class of cases.

Dr. John H. Packard, of Philadelphia, read a paper on "Supra-Pubic Cystotomy."

The present paper was supplementary to one on the same subject read by Dr. Packard at the last meeting, and was intended to correct an accidental omission of the views of Sir Henry Thompson. These were now considered, extensive quotations from the works of this author being given. Two cases were reported, in one of which the operation was performed for the removal of a portion of a silver catheter broken off in the bladder; and in the other, for the removal of a piece of rubber catheter, said to have been broken off several months previously. In this case, a stone weighing five hundred and seventy-one grains was removed, in the interior of which was found the *foreign body*.

Professor S. W. Gross, of Philadelphia, thought that Dr. McGuire was to be congratulated on having introduced a new operation, based upon the mechanism of the bladder, and the physiology of mictu-rition—the formation of an artificial uretha in a new position. The various operations which had been performed for the relief of prostatic obstruction were next referred to, the operations of Harrison, Mercier, Bartinini, and McGill being considered.

Professor WILLIAM T. BRIGGS, of Nashville, thought that perhaps in the cases reported by Dr. McGuire, a better result might have been obtained by lateral lithotomy, provided the stones were not too large to be removed by that route, for he had noted that after incision into the prostate, the gland diminished in size.

Mr. REGINALD HARRISON, of Liverpool, England, said that there are two general methods of relieving obstruction due to enlarged prostate, one by attacking the gland through the bladder, and the other through the perineum. In his operation he makes a median or lateral incision through the perineum according to circumstances. The obstructing prostate is next divided with considerable freedom, and a drainage-tube of considerable size introduced. From this operation he gets good results. Perineal lithotomy is preferable to the supra-pubic operation, because the lateral incision gives sufficient room for all manipulations. It gives an ample opening for the removal of a stone of considerable dimensions. It also permits of the more or less permament drainage which these cases require. He had also, through a perineal opening, used the perineal lithotrite with success. All methods of operation should be remembered, and each employed in those cases where it seems indicated.

Professor THOMAS ANNANDALE, of Edinburgh, Scotland, had come to the conclusion that if an operation is to be performed for the relief of prostatic obstruction, the perineal operation is the best. This allows of examination of the bladder, permits drainage, and probably causes a diminution of the hypertrophy. It enables you to occasionally remove a portion of the enlarged prostate when this assumes a pedunculated form. The speaker exhibited a rubber tube which he had found useful in cases of Harrison's operation when a permanent tube is required.

Mr. ARTHUR DURHAM, of London, England, emphatically indorsed what had been said in regard to operation for stone, that no one operation is applicable to all cases. It is a great mistake to be men of one method, especially in surgery. When we hear a man say that he treats all his cases of fracture in such a way, all his cases of stone in such a way, and all his cases of prostatic disease in such a way, we may be sure that such a man has a very small practice and experience, or else is a very great fool. In cases in which stone in the bladder is complicated with enlarged prostate, the perineal incision seems to me to be better than the supra-pubic.

Professor HINGSTON, of Montreal, Canada, believed that the supra-pubic operation was one to be performed only in exceptional cases. These he classified as follows: 1. In those cases of stricture in which the obstruction cannot be overcome in time to relieve the patient of great suffering. 2. In cases of prostatic obstruction. 3. In cases of tumors of the bladder which would interfere with the lateral operation. 4. In cases where the stone is too hard or too large to be removed either by lithotrity or by lateral lithotomy. He had himself removed, without injury to the soft parts, a stone weighing five ounces and five drachms.

Sir WILLIAM MACCORMAC, of London, England, had performed the operation of supra-cystotomy occasionally. He had never seen any untoward consequences, and the operation seems to be devoid of all risk. He did not consider that drainage was necessary after this operation. The bladder empties itself freely and the drainage-tube is a source of irritation.

The President, Professor D. HAYES AGNEW, being called upon, said the ground had been so thoroughly covered that there was little to be added. He agreed with those who held that no single operation was applicable to all cases. The supra-pubic operation may be appropriate in certain cases, while in others the perineal operation is the proper one. In order to avoid the unpleasant consequences which occasionally follow the perineal operation in children, where the prostate is small, I avoid the introduction of the finger into the bladder, and remove the stone with forceps not much larger than the staff.

While there may be no danger in the supra-pubic operation in skilled hands, yet with the inexperienced operator there will be risk of opening the peritoneal cavity. He thought that a number of cases had been reported of rupture of the bladder following injection of the organ after dilatation of the rectum with the rubber-bag.

The following are the officers of the Association for the following year: President, Dr. D. W. Cheever, of Boston; Vice-Presidents, Dr. T. W. Richardson, of New Orleans, and Dr. John B. Roberts, of Philadelphia; Secretary, Dr. J. R. Weist, Richmond, Indiana; Treasurer, Dr. P. S. Conner, of Cincinnati; Recorder, Dr. J. Ewing Mears, of Philadelphia; Additional Members of Council, Dr. W. F. Peck, of Davenport, and Dr. S. W. Gross of Philadelphia.

The next meeting to be held, beginning the second Tuesday in May, 1889, at Washington.

Dr. William T. Bull, of New York, presented a communication on "The Surgical Management of Typhlitis and Perityphlitis."

Dr. J. Ewing Mears, of Philadelphia, read a paper on "The Propriety of Surgical Interference in Perforating Typhoid Ulcer."

### Third Day.

Dr. George W. Gay, of Boston, read a paper entitled "The Comparative Methods of Tracheotomy and Intubation in the Treatment of Croup," of which the following were the conclusions :

1. Intubation may be tried in all cases of croup.

2. It is preferable in young children and in cases in which the tube must be left entirely to itself.

3. It may be resorted to for euthanasia provided the operator is reasonably expert and can do it without producing collapse.

4. Tracheotomy is called for in those cases in which intubation can not be done, or in which it fails to give relief, or in which the laryngeal tube is repeatedly rejected, or requires frequent removal for cleansing. It may also be required in those cases in which sufficient food cannot be given while the O'Dwyer tube is in position. It is also preferable in cases situated at a distance from a surgeon capable of introducing the laryngeal tube.

5. The tracheotomy instruments should always be at hand in intubation in case of emergency.

Dr. H. H. Mudd, of St. Louis, said that intubation had been done as a precautionary measure in many cases in which tracheotomy would not have been thought of. Some of the good results of intubation are to be attributed to this fact. In most of his cases of intubation where the patient survived he had found it necessary to resort to tracheotomy ; patients have recovered after tracheotomy where intubation has proved unsuccessful.

Professor Thomas Annandale called attention to the value of the introduction of a tube through the glottis in cases of operation about the throat where there was risk of suffocation or of hæmorrhage into the trachea.

Dr. Huber, of New York, had performed intubation in ninety-four cases with recovery in thirty-seven. He does not operate early. He considers the internal use of bichloride of mercury as of equal importance as the intubation. There is occasionally an advantage in using a small tube with the expectation that it will be coughed out, and with it a portion of the membrane, and affording an opportunity for feeding while the tube is out.

Dr. T. F. PREWITT, of St. Louis, in one case of diphtheric paralysis, had, in order to avoid passage of fluid into the larynx, passed a catheter through the glottis and plugged the larynx with a sponge. This permitted the fluid to go into the œsophagus without risk of entering the trachea. After feeding, the sponge and tube were removed.

Dr. D. W. CHEEVER, of Boston, advocated the disuse of anæsthetics in cases of tracheotomy, provided proper assistants can be secured. The operation is not accompanied with much pain. By avoiding the anæsthetic many of the risks of the operation are avoided.

Dr. L. McLANE TIFFANY, of Baltimore, read a paper entitled "Pregnancy and Operative Surgery—their Mutual Relations," of which the following were the conclusions:

1. Pregnancy is a physiological condition and does not contra-indicate a surgical operation.

2. During pregnancy temporary strain may be exerted on some organ, *e. g.*, kidney, inducing impairment of function.

3. A surgical operation upon a pregnant woman is to be conducted so as to avoid inducing abortion, in itself a serious accident.

4. The main cause of abortion after operation is sepsis.

5. The probability of sepsis after operation is increased if the patient is suffering from disease, either temporary or chronic.

6. Abortion may result from operation —shock, perhaps.

7. Hæmorrhage does not seem to induce abortion.

8. Union of fracture may be retarded by pregnancy.

9. Recorded cases show that the unborn child receives no evil impress when the mother is subjected to operation.

10. When a surgical operation upon a pregnant woman is under consideration, the function of all the patient's organs must be carefully investigated and regulated. An operation then conducted antiseptically may be expected to result as though pregnancy were not present.

Dr. J. EWING MEARS, of Philadelphia, thought that while pregnancy was to be be regarded as a physiological process in the native woman, it could not be considered in this light in the society woman. Another important point to be considered was whether the operation required was one of expediency or of necessity. In the latter case the surgeon must do his duty, let the result be what it may, but whether or not operations of expediency were to be performed on the pregnant woman was a question only to be decided by further experience.

Professor P. S. CONNER, of Cincinnati, reported a case of "Subcutaneous Operation for Anchylosis of the Knee," in a woman who, it was subsequently learned, was six weeks pregnant. The operation was followed by severe septic infection, but this did not interfere with the normal course of the pregnancy.

Dr. WILLIAM HUNT, of Philadelphia, raised the question whether or not in the case of inevitably fatal injury of a pregnant woman, as from burns, it was justifiable to run the risk of sacrificing the mother a few days sooner, when by so doing the life of the child might be saved, or must we wait until the last breath has left the body before making the incision.

Dr. R. B. BONTECOU, of Troy, reported the case of a rupture of an umbilical hernia in a woman seven months pregnant. The intestines were out four hours. They were then cleaned and replaced. The woman recovered and a healthy child was born one month later.

Dr. L. McLANE TIFFANY, of Baltimore, thought that in the case suggested by Dr. Hunt there could be no question as to the propriety of operation. By so doing one life may be saved.

Dr. N. P. Dandridge, of Cincinnati, read a paper on "Nerve-Stretching."

The following conclusions were presented:

1. That nerve-stretching should be condemned in all forms of central disease, such as tabes, myelitis, etc.

2. That it offers little prospect of relief in tetanus.

3. That it should be regarded as a reliable method in cases of persistent neuralgia and peripheral paralysis of sensation in the extremities.

4. That stretching the facial is indicated in tic-convulsif.

5. That further trial is justified in reflex epilepsy.

6. That stretching the lingual should be tried in painful affections of the tongue.

7. That resection should always be preferred to stretching in the spinal accessory and in the branches of the fifth nerve except the lingual.

Dr. De Forest Willard, of Philadelphia, read a report of three cases of Nephrectomy performed for — 1, gunshot wound of the kidney; 2, tubercular disease of the kidney.

